# Rescuing activity of oxygen-damaged pyruvate formate-lyase by a spare part protein

**DOI:** 10.1016/j.jbc.2021.101423

**Published:** 2021-11-18

**Authors:** Mary C. Andorfer, Lindsey R.F. Backman, Phoebe L. Li, Emily C. Ulrich, Catherine L. Drennan

**Affiliations:** 1 Department of Biology, Massachusetts Institute of Technology, Cambridge, Massachusetts, USA; 2 Howard Hughes Medical Institute, Massachusetts Institute of Technology, Cambridge, Massachusetts, USA; 3 Department of Chemistry, Massachusetts Institute of Technology, Cambridge, Massachusetts, USA; 4 Center for Environmental Health, Massachusetts Institute of Technology, Cambridge, Massachusetts, USA

**Keywords:** radical chemistry, enzyme inactivation, electron paramagnetic resonance (EPR) spectroscopy, isothermal titration calorimetry (ITC), protein complex, glycyl radical enzyme, cofactor repair, spare part protein, oxygen-sensitive enzymes, bacterial metabolism, AE, activating enzyme, cPFL, oxygen-cleaved PFL, GcrA, autonomous glycyl radical cofactors, GRE, glycyl radical enzyme, PFL, pyruvate formate-lyase, tPFL, truncated PFL, truncYfiD, truncated YfiD

## Abstract

Pyruvate formate-lyase (PFL) is a glycyl radical enzyme (GRE) that converts pyruvate and coenzyme A into acetyl-CoA and formate in a reaction that is crucial to the primary metabolism of many anaerobic bacteria. The glycyl radical cofactor, which is posttranslationally installed by a radical *S*-adenosyl-L-methionine (SAM) activase, is a simple and effective catalyst, but is also susceptible to oxidative damage in microaerobic environments. Such damage occurs at the glycyl radical cofactor, resulting in cleaved PFL (cPFL). Bacteria have evolved a spare part protein termed YfiD that can be used to repair cPFL. Previously, we obtained a structure of YfiD by NMR spectroscopy and found that the N-terminus of YfiD was disordered and that the C-terminus of YfiD duplicates the structure of the C-terminus of PFL, including a β-strand that is not removed by the oxygen-induced cleavage. We also showed that cPFL is highly susceptible to proteolysis, suggesting that YfiD rescue of cPFL competes with protein degradation. Here, we probe the mechanism by which YfiD can bind and restore activity to cPFL through enzymatic and spectroscopic studies. Our data show that the disordered N-terminal region of YfiD is important for YfiD glycyl radical installation but not for catalysis, and that the duplicate β-strand does not need to be cleaved from cPFL for YfiD to bind. In fact, truncation of this PFL region prevents YfiD rescue. Collectively our data suggest the molecular mechanisms by which YfiD activation is precluded both when PFL is not damaged and when it is highly damaged.

Glycyl radical enzymes (GREs) are a growing superfamily that allows microbes to perform challenging chemistry anaerobically (1). Certain GREs, such as class III ribonucleotide reductase (2) and the toluene-metabolizing enzyme benzyl succinate synthase (3), have been studied for decades, whereas many GREs are currently being discovered and characterized for the first time (4). Newly characterized GRE function varies greatly, with known roles in *trans*-4-hydroxy-L-proline metabolism (5, 6), sulfite acquisition (7, 8, 9), and toluene synthesis (10). The best-studied GRE, pyruvate formate-lyase (PFL) from *Escherichia coli*, converts pyruvate and CoA into formate and acetyl-CoA through a proposed mechanism involving glycyl and thiyl protein-based radical intermediates (Fig. 1), which is channeled into anaerobic metabolism (1, 11, 12). Due to this function in primary anaerobic glucose metabolism, PFLs are prevalent within the human gut microbiome (5). In addition to the medical significance of gut microbiome enzymes, GREs hold promise for an array of industrial applications, including the production of value-added chemicals (13, 14, 15) and degradation of environmental pollutants (14, 16). PFL serves as such an example; it has been shown that the reverse reaction can be performed *in vivo*, thus opening up the possibility of using this abundant metabolic enzyme in acetate assimilation and/or formate fixation (15). Additionally, the GRE glycerol dehydratase provides a route for the inexpensive production of propane-1,3-diol—an important monomer for plastic and lubricant synthesis—from glycerol, a by-product of biodiesel manufacturing (13).

**Figure 1:**
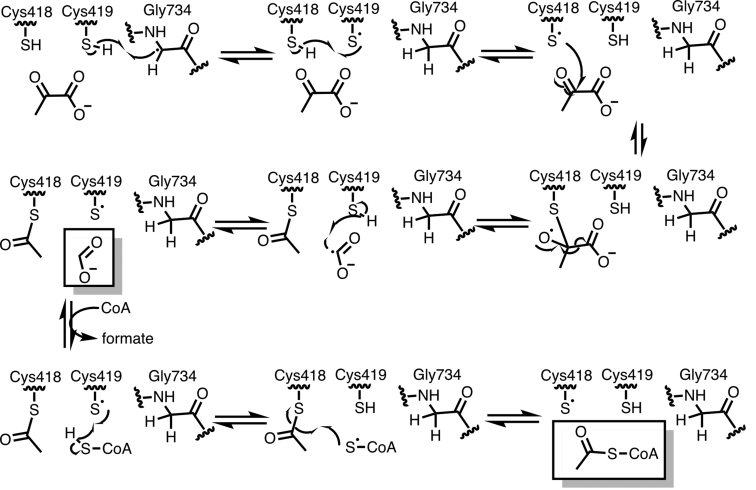
**Proposed mechanism of PFL**.

Enabling the challenging chemistry of GRE enzymes (Fig. 2) is a common glycyl radical cofactor. This glycyl radical cofactor is installed on a backbone glycine residue within the GRE by a radical *S*-adenosyl-L-methionine (SAM, AdoMet) dependent activating enzyme (AE) (Fig. 3*A*) (17, 18). The details of how this activation step is accomplished are largely unknown; however, based on structural and biochemical data, it is thought that conformational changes are required (19, 20). Briefly, a loop within the GRE containing the catalytic glycine residue is thought to flip out of the buried active site, bind inside AE’s active site where the glycyl radical is installed, and flip back into the GRE where it is protected by a 10-stranded β-barrel further surrounded by α-helices (Fig. 3*B*). Once the glycyl radical is formed, it can generate a transient thiyl radical on a nearby cysteine residue (Fig. 3, *A,* i), also buried deep inside the 10-stranded barrel. This thiyl radical abstracts a hydrogen atom from substrate to initiate catalysis (21, 22). Because the radical chemistry is highly controlled within the GREs, the glycyl radical can persist for several days *in vitro* (12, 23) and catalyze numerous turnovers from one initial activation step, making it an attractive catalyst. The enzyme architecture is thought to protect the radical cofactor from being quenched under anaerobic conditions (1); however, it is unable to protect against oxygen exposure, making GREs oxygen-sensitive enzymes.

**Figure 2:**
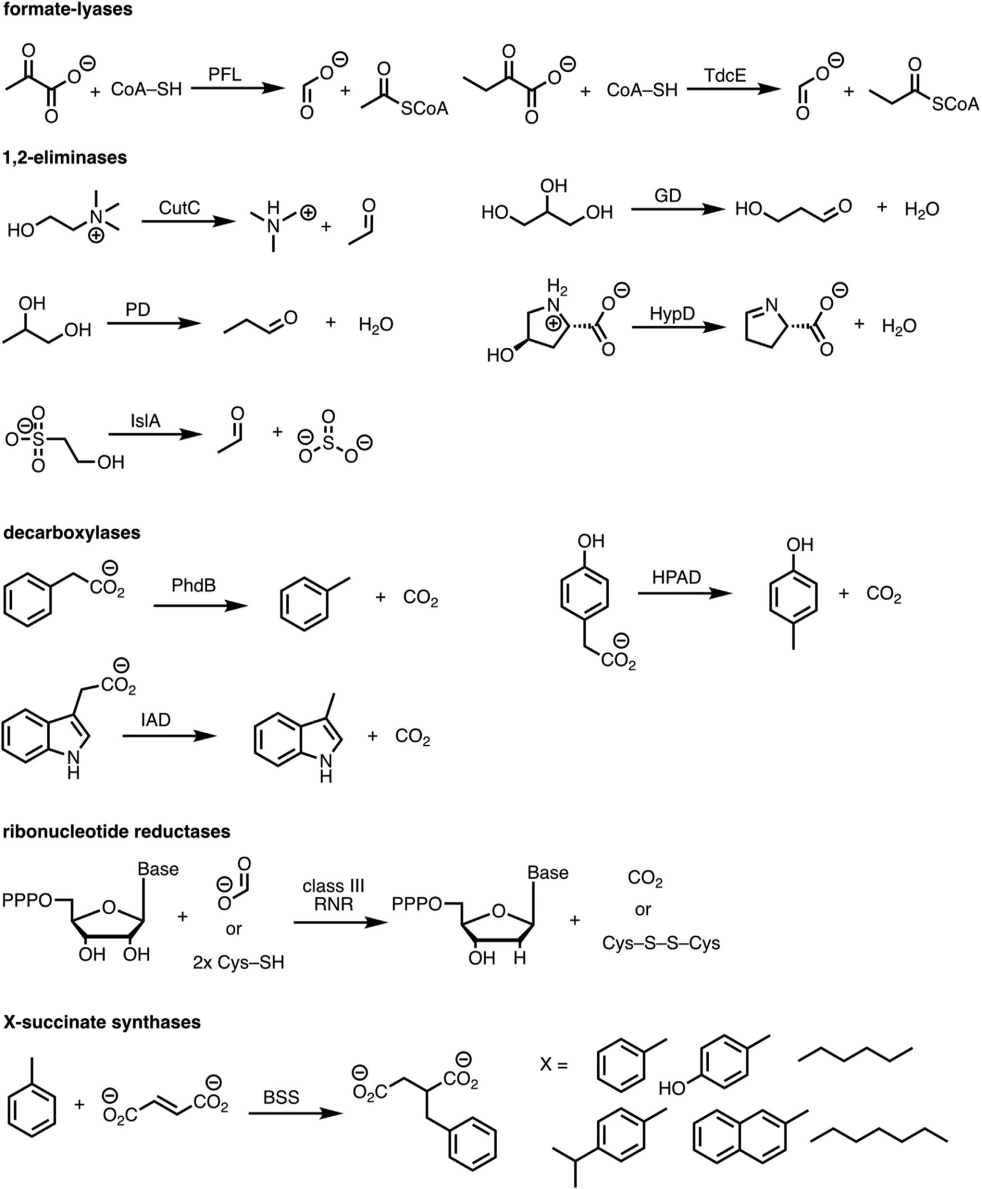
**GREs catalyze a wide variety of challenging reactions using radical chemistry**.

**Figure 3:**
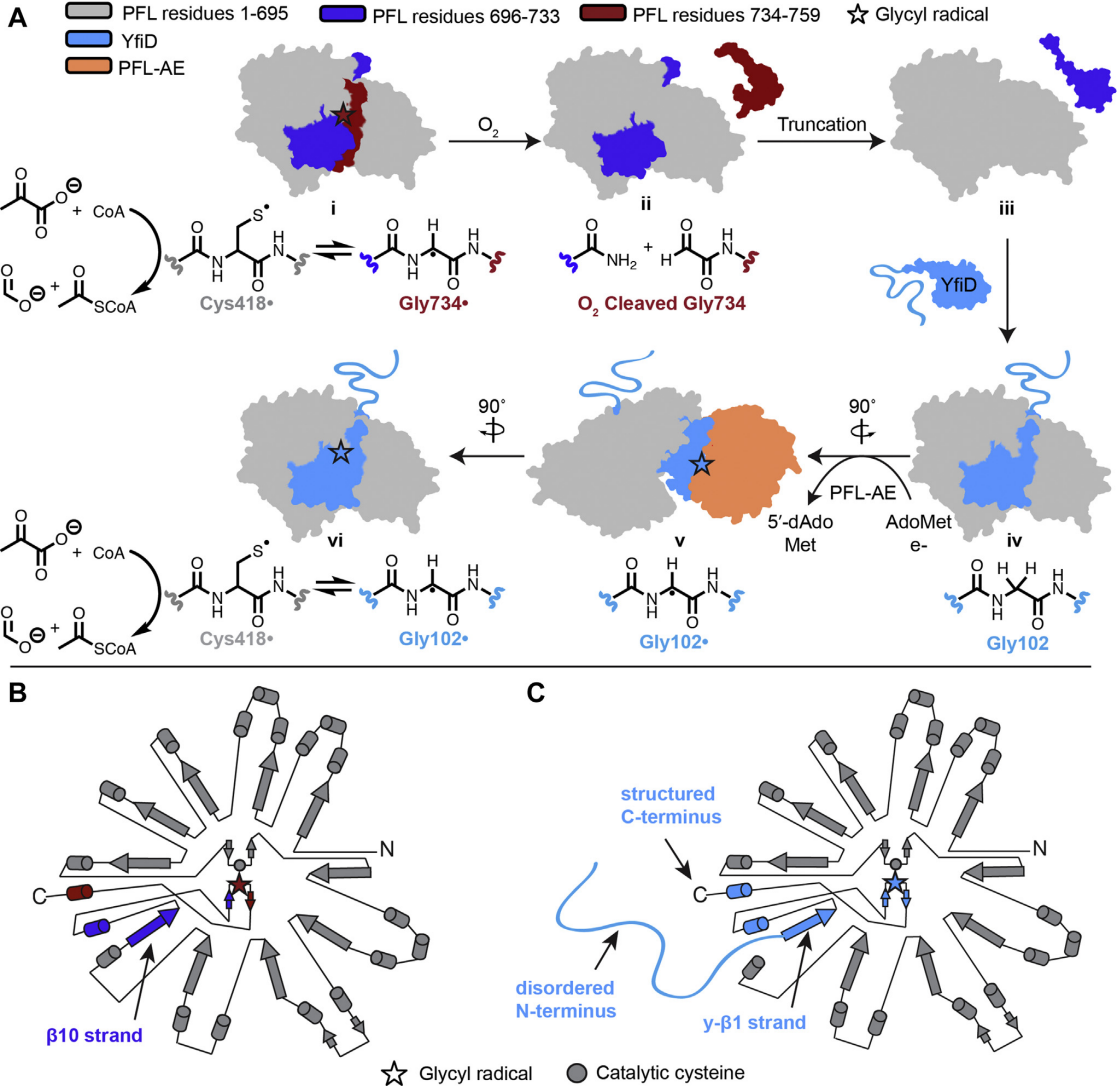
**Initial model for O**_**2**_**-damaged PFL rescue of activity by YfiD and topology diagrams.***A*, crystal structures of PFL and PFL-AE (PDB ID: 2PFL and 3CB8, respectively) and NMR structure of YfiD (PDB ID: 6OWR) were used to create cartoons. No structural data are available for any of the above protein complexes—cartoons of complexes were created by manually docking structures as previously described. Color coding is as follows: PFL residues 1 to 695 in *gray*, PFL residues 696 to 733 in *dark blue*, PFL residues 734 to 759 in *red*, PFL-AE in *orange*, YfiD in *light blue*. *B*, topology diagram of PFL with residues 1 to 695 in *gray*, 696 to 733 in *dark blue*, and 734 to 759 in *red*. *C*, topology diagram of truncated PFL (tPFL, *gray*) in complex with YfiD (*light blue*). Residues 1 to 60 of YfiD are disordered in the NMR structure and residues 61 to 127 of YfiD have the same fold as residues 693 to 759 of PFL, which includes a β strand (β10 in PFL and y-β1 in YfiD), the glycyl radical loop, and a C-terminal helix.

The damage to GREs from molecular oxygen is severe and has limited their industrial use (13, 14, 24). The damage is caused by the ability of molecular oxygen to diffuse into the enzyme active site and react with the glycyl radical, ultimately leading to peptide cleavage at the site of the glycyl radical, G734 in *E. coli* PFL, generating cleaved PFL (cPFL) (Fig. 3*A*, ii) (21, 25, 26). Many GREs are found within facultative anaerobes (*i.e.*, bacteria that can survive anaerobically as well as aerobically) for which exposure to microaerobic conditions can occur. Knappe and coworkers discovered a unique repair mechanism for the constitutively expressed PFL; both *E. coli* and bacteriophage T4 constitutively produce small (∼14 kDa) “spare part” proteins (YfiD in *E. coli* and Y06I in T4) that restore functionality to a ∼170 kDa O_2_-damaged cPFL (Fig. 3*A*) and thus allow organisms to overcome some of the challenges associated with GRE oxygen exposure (27). Restoration of PFL activity involves glycyl radical installation on YfiD or Y06I by the PFL radical SAM activating enzyme (PFL-AE) and complex formation between the activated YfiD/Y06I and the oxygen-cleaved cPFL (27, 28). To date, only these two spare part proteins for PFL have been validated; however, because all GREs are susceptible to this mechanism of oxygen damage, it is possible that spare part proteins exist for other GREs as well.

We have recently proposed a mechanism for rescue of activity of O_2_-damaged *E. coli* PFL (UniProt ID: P09373) by YfiD (UniProt ID: P68066) and PFL-AE (UniProt ID: P0A9N4) (Fig. 3*A*) based primarily on structural data (28). Through structural characterization of the 14.3 kDa YfiD by nuclear magnetic resonance (NMR) spectroscopy, we have shown that there are two domains of YfiD: the disordered N-terminal half (residues 1–60) and the structured C-terminal half (residues 61–127) (Fig. 3*C*). The C-terminus of YfiD has high sequence and structural similarity to the portion of PFL that is cleaved upon oxygen exposure and is the domain that binds in the active site of PFL and harbors the glycyl radical. Based on docking models, we proposed that cPFL, which is cleaved at the glycyl radical at position 734, must be further truncated to tPFL (residues 696–733 removed) to allow the C-terminal structured domain of YfiD to bind (Fig. 3*A*, iii). This truncation results in loss of one of the β-strands from the 10-stranded barrel of PFL, which we propose is replaced by the y-β1 strand from the C-terminus of YfiD (Fig. 3, *B* and *C*) (28). We have further proposed that glycyl radical installation on YfiD by PFL-AE occurs *after* YfiD binds to cPFL (Fig. 3*A*, iv to v) (28), which would prevent glycyl radical quenching prior to YfiD association with cPFL and would also protect the cell from unwanted radical chemistry.

Chemical logic would dictate that for “spare part rescue” to function efficiently and safely in a cell, there must be molecular mechanisms in place to regulate glycyl radical installation and GRE target binding. Here we probe the fascinating and unique oxygen rescue mechanism of a GRE by a spare part protein using isothermal titration calorimetry (ITC), electron paramagnetic resonance (EPR) spectroscopy, and kinetic analyses. Although sources of oxygen damage and repair in oxygen-sensitive, industrially relevant metalloproteins have been studied in the past (13, 29, 30, 31), mechanisms proposed for spare part rescue of GREs have not been experimentally tested. In this work, we attempt to understand how the cell is protected from a radical-containing spare part protein and also how such a spare part is directed toward a minimally damaged GRE that can be rescued while prevented from associating with a GRE that is too heavily damaged for repair. An understanding of these mechanisms will provide insight into biological modes of enzymatic repair, into microbial community survival in microaerobic environments, and also inform future efforts for the engineering of spare parts for other industrially relevant GREs.

## Results

**Figure 4:**
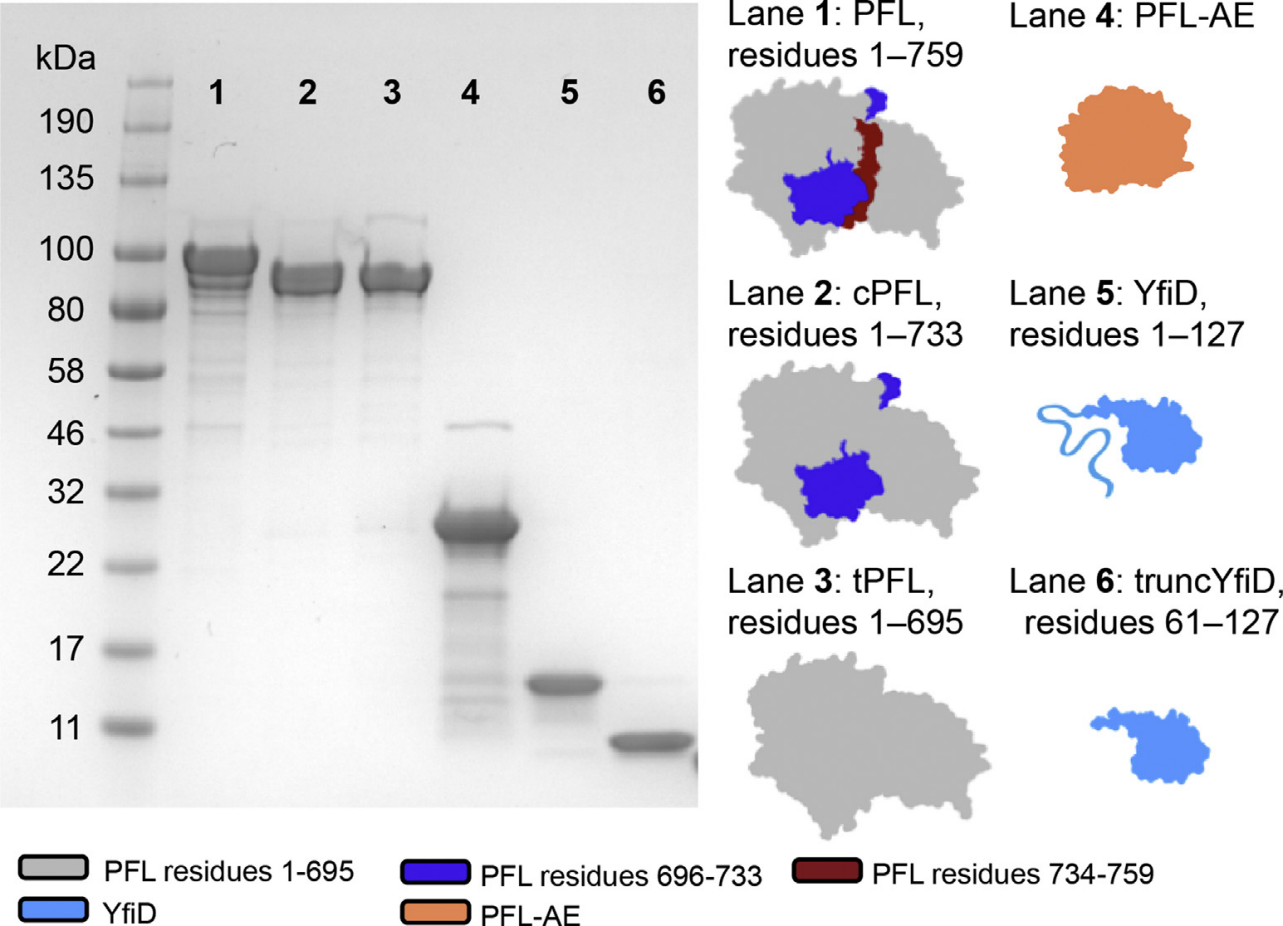
**Summary of constructs.** Gel, cartoons, names, and construct length for all proteins used in this study.

We probed the mechanism of oxygen-damaged GRE repair by employing truncation variants of PFL and YfiD (Fig. 4). Previously, we had made a mimic of oxygen-damaged PFL by replacing Gly734 with a stop codon (28). The resulting cPFL protein only differs from O_2_-damaged PFL in that the C-terminus ends in a carboxylic acid instead of an amide (Fig. 3*A*, ii). To examine whether YfiD binding requires a further proteolysis event in which cPFL (residues 1–733) is further shortened to tPFL (residues 1–695) (Fig. 3*A*, ii to iii), we also made a tPFL construct that replaces residue E696 with a stop codon. To investigate the role of YfiD’s disordered N-terminus in cPFL rescue, we used a truncated YfiD construct (residues 61–127) that lacked the disordered N-terminus (truncYfiD) (28). All of these constructs were able to be readily overexpressed, purified, and used for biochemical experiments to probe the roles of targeted regions of PFL and YfiD (Fig. 4).

### The N-terminus of YfiD is important for activation by PFL-AE but does not affect enzyme activity

**Figure 5:**
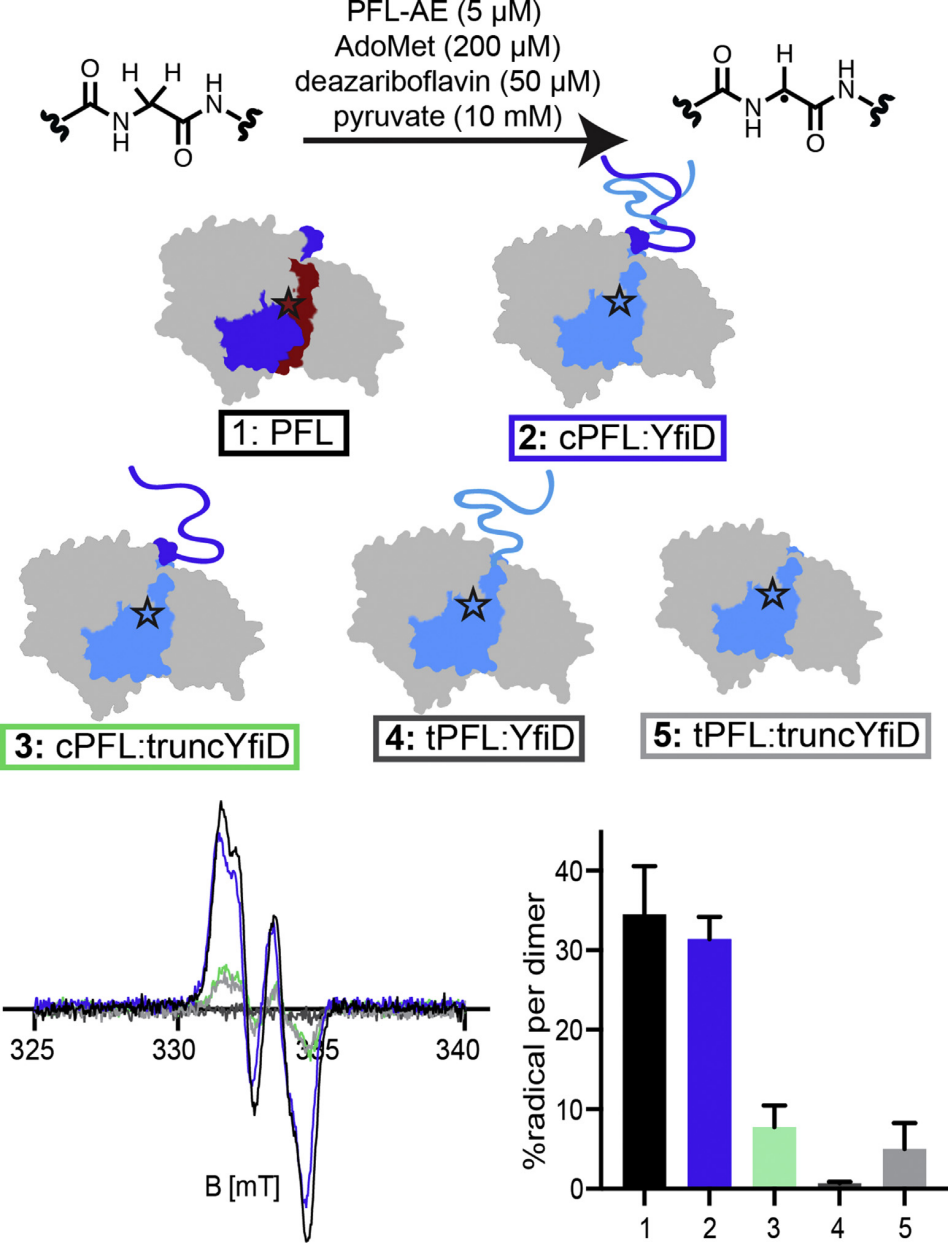
**Glycyl radical installation comparisons.***Top*: Reaction conditions for activations of PFL and PFL:YfiD complexes. *Middle*: Cartoon representations of PFL and PFL:YfiD complexes. *Bottom*: EPR data used to quantify amounts of glycyl radical (N = 3). Briefly, in an anaerobic chamber, PFL variants (200 μM final conc.) and YfiD variants (200 μM final conc.) were diluted with 20 mM HEPES pH 7.2 to a final volume of 150 μl. Pyruvate, PFL-AE, AdoMet, and 5-deazariboflavin were added to each reaction. Activation buffer was added to each reaction for a final volume of 300 μl. The activations were mixed by pipetting and illuminated using a 500 W halogen lamp for 15 to 30 min. EPR spectroscopy was used to quantify glycyl radical content. EPR parameters were as follows: 80 K, 9.37 GHz, modulation amplitude of 3 G, microwave power of 1.26 μW.

The tPFL:truncYfiD docking model has the same architecture as wild-type PFL, where truncYfiD is sufficient to fully replace PFL residues 734 to 759 and 696 to 733 that are lost upon oxygen cleavage and subsequent truncation, respectively (Fig. 3). This observation led us to question the role and importance of YfiD’s disordered N-terminus (residues 1–60). To probe the role (if any), we employed the truncYfiD construct that is lacking the N-terminus (residues 1–60) and cPFL, which is missing residues 734 to 759 that are cleaved upon oxygen exposure (28). We wanted to compare the efficiency of glycyl radical installation on the cPFL:YfiD complex to that of the cPFL:truncYfiD complex. We used protocols similar to those previously published for activation reactions and quantified glycyl radical incorporation by EPR spectroscopy (Fig. S1, Table S1) (18). A comparable amount of the radical is installed on PFL and cPFL:YfiD, the two wild-type systems (17.2 μM and 15.9 μM, respectively, Fig. 5, **1** and **2**). Without the N-terminus of YfiD, the activation levels are nearly fourfold lower (15.9 μM for cPFL:YfiD drops to 4.1 μM for cPFL:truncYfiD, Fig. 5, **2** and **3**). This finding indicates that although the N-terminus is not necessary for activation, removing this region of YfiD decreases the glycyl radical signal. No detectable radical was observed for any of the individual components of complexes **2** or **3** individually, demonstrating that the EPR signal is not arising from native PFL and YfiD contaminants.

**Figure 6:**
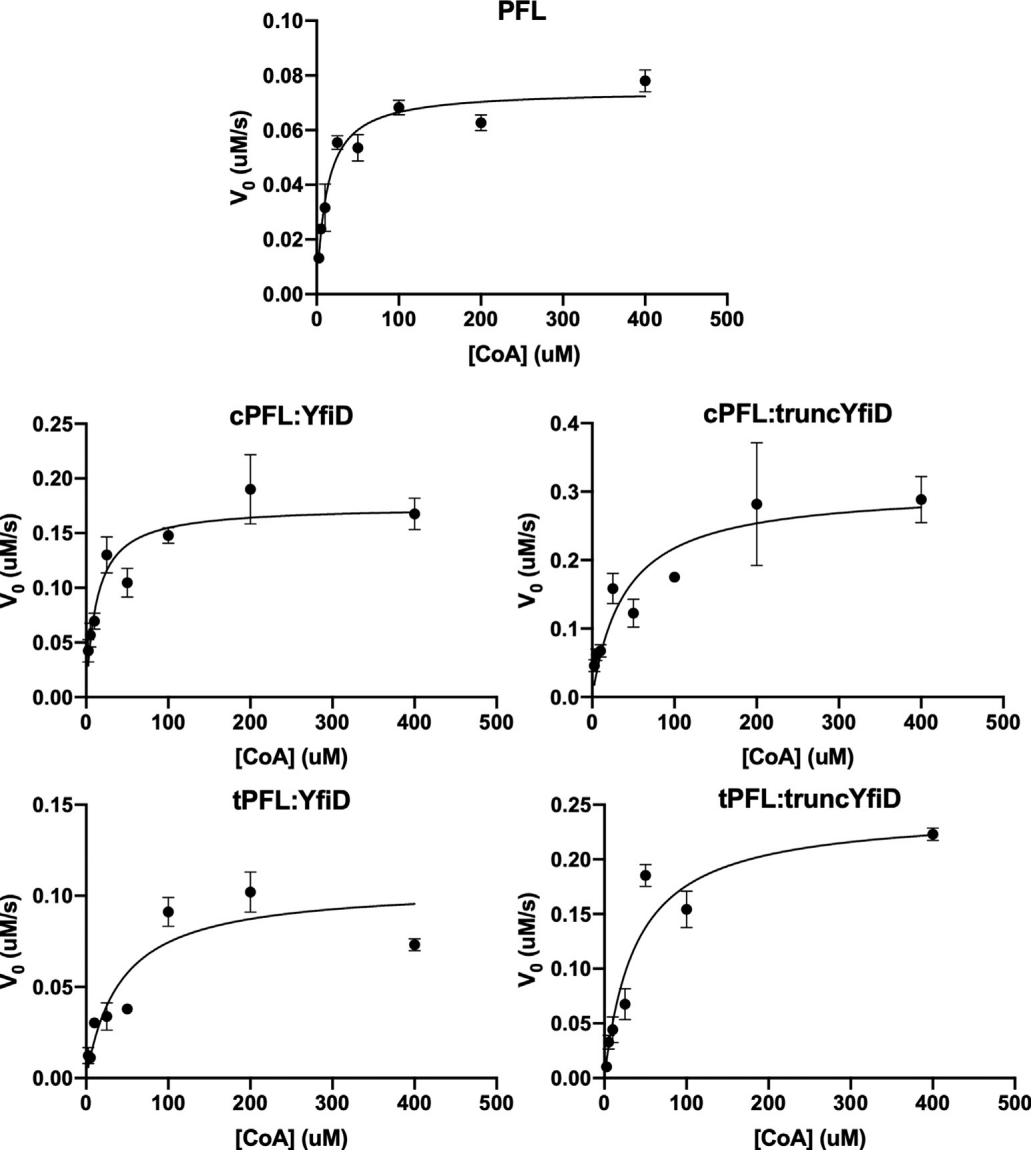
**Saturation plots for wild-type PFL and four PFL:YfiD complexes.** Production of acetyl-CoA by PFL and PFL:YfiD complexes was measured through a coupled assay with citrate synthase and malic acid dehydrogenase. Inside of an anaerobic chamber, citrate synthase (6 U per reaction), malic acid dehydrogenase (14 U per reaction), CoA (2.5–400 μM) were added to assay buffer (150 mM Tris pH 8.5, 10 mM L-malate, 10 mM pyruvate, 3 mM NAD). Activated PFL or PFL:YfiD mixture was added to initiate the reaction and immediately pipetted to mix. Data were collected on an Ocean Optics Spectrometer at 366 nm to measure absorbance of NADH. Initial velocity curves were conducted in triplicate for each CoA concentration at 21 °C and plotted using Prism nonlinear regression software to calculate K_M_ and V_max_ for each complex. EPR spectroscopy was used to measure glycyl radical content for PFL and PFL:YfiD complexes, and the final concentrations of radical in reactions were used as E_tot_. V_max_ and E_tot_ were used to calculate k_cat_.

We determined the kinetic parameters of each complex for the conversion of pyruvate and CoA to formate and acetyl-CoA using coupled activity assays and UV-vis spectroscopy (11). The apparent K_M_ values of PFL, cPFL:YfiD, and cPFL:truncYfiD for CoA were found to be 12 ± 4, 13 ± 6, and 40 ± 26 μM, respectively (Table 1 and Fig. 6). The apparent k_cat_ values were calculated using the amount of activated PFL or activated complex, as determined by EPR spectroscopy, as opposed to the total amount of protein. The apparent k_cat_ values for PFL, cPFL:YfiD, and cPFL:truncYfiD were found to be 105 ± 7, 130 ± 14, and 186 ± 36 s^−1^, respectively (Table 1 and Fig. 6). (Note that apparent k_cat_ values are lower than previously reported for PFL (770 s^−1^) (11). This variation is likely attributed to differences in experimental setup, one being temperature.) From these experiments, it appears that removing the N-terminal half of YfiD does not substantially change the catalytic efficiency of acetyl-CoA production (Table 1, entries **2** and **3**).

**Table 1:** Kinetic parameters of PFL and PFL:YfiD complexes

Protein(s)		K_M, app_ (μM)	k_cat, app_ (s^−1^)	E_tot_ (μM)^a^	V_max_ (μM s^−1^)
1	PFL	12 ± 4	105 ± 7	0.000714	0.075
2	cPFL:YfiD	13 ± 6	130 ± 14	0.00134	0.1745
3	cPFL:truncYfiD	40 ± 26	186 ± 36	0.00164	0.3048
4	tPFL:YfiD	42 ± 27	121 ± 25	0.00087	0.1055
5	tPFL:truncYfiD	39 ± 18	204 ± 32	0.001194	0.2437

a E_tot_ refers to the final concentration of glycyl radical in reactions.

### Residues 696 to 733 of cPFL are important for YfiD activation by PFL-AE but do not impact enzyme activity of the rescued PFL:YfiD complex

Based on structural data (28), the C-terminus of cPFL (residues 696–733) must be moved out of the active site to make room for YfiD to bind so that YfiD’s β-strand can occupy the position of PFL’s β10 strand (Fig. 3, *B* and *C*). This observation has led us to propose a truncation of cPFL by proteases to remove residues 696 to 733 (Fig. 3*A*, iii). We used our tPFL construct (Fig. 4), which lacks these residues of the C-terminus of cPFL to investigate this hypothesis. We again conducted activation reactions for both the tPFL:YfiD and the tPFL:truncYfiD complexes and quantified glycyl radical incorporation by EPR spectroscopy. We observe a 32-fold decrease in activation of the tPFL:YfiD complex compared with cPFL:YfiD (15.9 μM and 0.5 μM for cPFL:YfiD and tPFL:YfiD, respectively, Fig. 5, complexes **2** and **4**). The 696 to 733 region of cPFL thus appears to play a crucial role in either PFL:YfiD binding or/and activation. Surprisingly, the negative effects on activation of these two different truncations (N-terminus of YfiD and residues 696–733 of PFL) are not additive. Instead, tPFL:truncYfiD has a 5.4-fold higher activation level than tPFL:YfiD (2.7 μM and 0.5 μM for tPFL:truncYfiD and tPFL:YfiD, respectively, Fig. 5, complexes **5** and **4**). No detectable radical was observed for any of the individual components of complexes **4** or **5** individually, demonstrating that the EPR signal is not arising from native PFL and YfiD contaminants. It is notable that wild-type YfiD cannot be activated without a PFL variant present in the mixture, supporting the proposal that YfiD binds to PFL before activation (27, 28).

The kinetic parameters of the tPFL:YfiD and tPFL:truncYfiD complexes were determined using the methods described above. The apparent K_M_ values of tPFL:YfiD and tPFL:truncYfiD for CoA were found to be 42 ± 27 and 39 ± 18 μM, respectively (Table 1 and Fig. 6). The apparent k_cat_ values for tPFL:YfiD and tPFL:truncYfiD were found to be 121 ± 25 and 204 ± 32 s^−1^, respectively (Table 1 and Fig. 6). From these experiments, it appears that residues 696 to 733 of cPFL do not contribute substantially to catalytic efficiency of acetyl-CoA production (Table 1, entries **4** and **5**).

### Residues 696 to 733 of cPFL and residues 1 to 60 of YfiD impact PFL:YfiD complex formation

Activation reactions used to initially assess glycyl radical installation were conducted using PFL and YfiD variants in a 1 PFL monomer:1 YfiD monomer ratio (Fig. 5). We wondered if higher concentrations of YfiD would affect the observed concentrations of glycyl radical incorporation. When we increase the concentration of YfiD in activation reactions from a 1:1 ratio of cPFL:YfiD to a 1:5 ratio, we observe a very small increase in glycyl radical concentration (11.9 ± 0.4 μM to 13.4 ± 0.6 μM, Fig. S2). A different effect was observed when we repeated this experiment for the tPFL:YfiD complex. When the ratio of tPFL:YfiD is increased from 1:1 to 1:5, we observe a fourfold increase in glycyl radical concentration (0.18 ± 0.06 μM to 0.75 ± 0.05 μM, Fig. S2).

**Figure 7:**
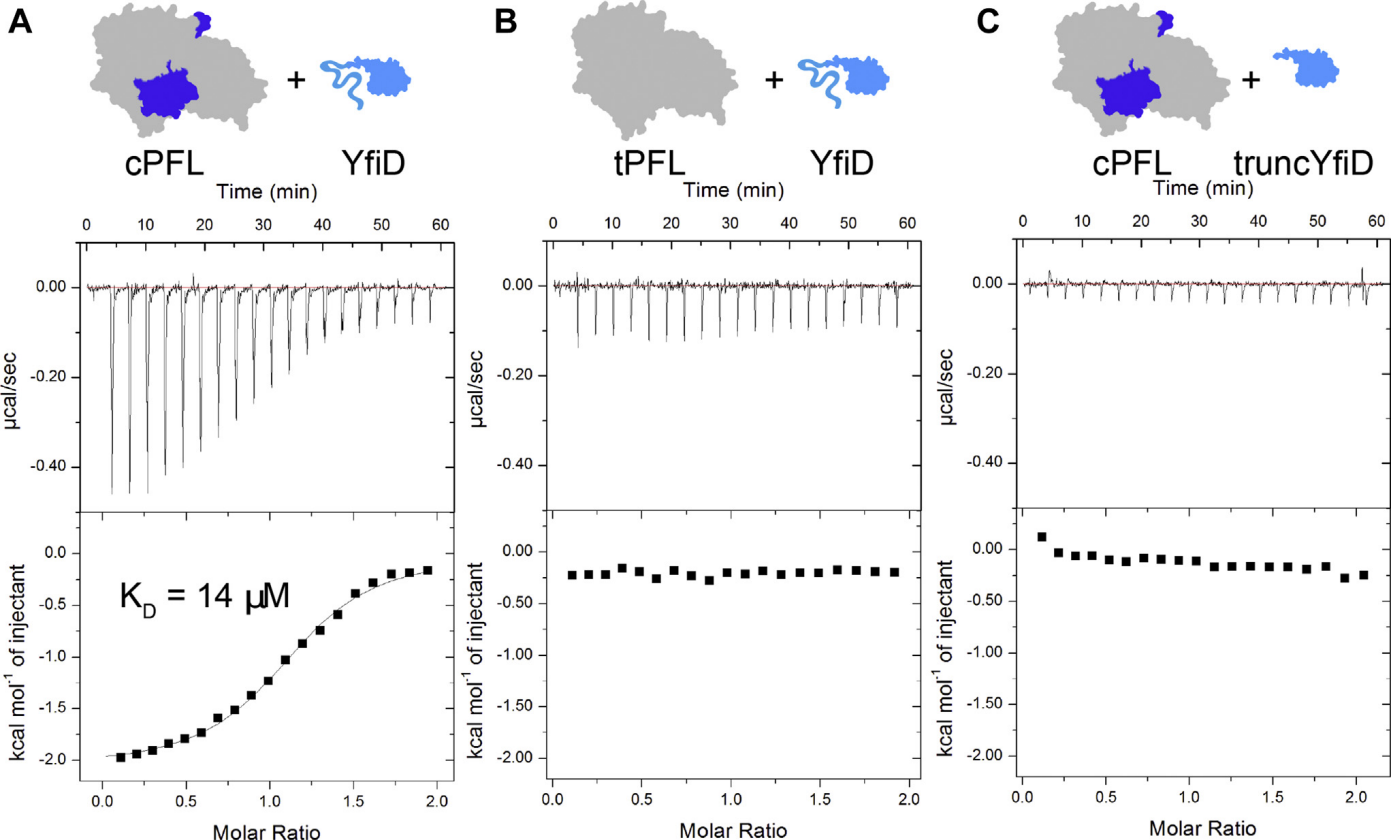
**ITC binding data for YfiD and truncYfiD added as titrant to cPFL and tPFL.***A*, an exothermic binding event between cPFL and YfiD occurs. The best fit is consistent with a K_D_ of 14 μM for the cPFL:YfiD complex. Initial [cPFL] in cell = 224 μM (186 μM final conc.), initial [YfiD] = 2.129 mM (361 μM final conc.). *B*, no clear binding event between tPFL and YfiD is observed. Instead, only the heat of dilution for YfiD can be observed. Initial [tPFL] in cell = 224 μM (186 μM final conc.), initial [YfiD] = 2.1 mM (356 μM final conc.). *C*, no clear, enthalpic binding event between cPFL and truncYfiD is observed. Instead, only the heat of dilution for truncYfiD can be observed. Initial [cPFL] in cell = 173 μM (143 μM final conc.), initial [truncYfiD] = 1.73 mM (294 μM final conc.).

Although this result could suggest that YfiD does not bind as well to tPFL as to cPFL, we wanted to directly compare binding between cPFL:YfiD and tPFL:YfiD. We employed isothermal titration calorimetry (ITC) and found that indeed, as we and others have hypothesized (27, 28), unactivated YfiD does bind to cPFL. When we titrate YfiD into a cell containing cPFL in matched buffer, the K_D_ was found to be 14 μM (Fig. 7*A*). We repeated the ITC experiments for tPFL:YfiD. The only difference between the two systems is the removal of residues 696 to 733 from cPFL to create the tPFL construct. At the concentrations used in these studies, we observe no enthalpic binding event between tPFL and YfiD (Fig. 7*B*). Given that residues 696 to 733 of cPFL contain a duplicate β-strand to that found in YfiD (Fig. 3, *B* and *C*), the former result is surprising. Similarly, when we titrate truncYfiD into a cell containing cPFL, no binding event detectable by ITC is observed (Fig. 7*C*, controls shown in Fig. S3, truncYfiD titrated into tPFL shown in Fig. S4). Thus, although the N-terminal half of YfiD is disordered in solution, it does appear to play a role in PFL:YfiD complex formation.

## Discussion

The idea of a spare part protein giving new life to an oxygen-damaged radical enzyme is compelling in terms of the metabolic expense of the cell synthesizing a 14-kDa protein instead of a 170-kDa one. The speed at which metabolic activity of PFL can be rescued is also attractive if the spare part protein is at the ready, *i.e.*, if the spare part protein is constitutively expressed. Notably, previous studies show that YfiD (32) and PFL-AE (33) are constitutively expressed in *E. coli*. However, if the activase and the spare part protein are both constitutively expressed, *how does the cell prevent activation of the spare part protein when no oxygen is around, which could lead to unwarranted radical chemistry?*

Previously, we proposed a possible answer to this question; that YfiD has the molecular equivalent of a safety lever in the form of a β-strand that hinders activation by PFL-AE in the absence of oxygen-cleaved PFL. This proposal was based on modeling studies that showed that a β-strand, y-β1, appears to block the glycyl residue of YfiD from binding close enough to the radical SAM cofactor of PFL-AE for radical generation (28). Supporting the idea that free YfiD is not an ideal substrate for PFL-AE, the K_M_ of YfiD for PFL-AE was estimated to be 100 μM (27), which is two orders of magnitude higher compared with the K_M_ of PFL-AE for PFL (1.4 μM) (34). We further proposed that activation of YfiD requires YfiD binding to cPFL and the subsequent displacement of YfiD’s y-β1 strand away from its activatable glycine. Since YfiD’s binding site on PFL does not exist when PFL is intact, this mechanism would ensure that YfiD is only activated after PFL is cleaved by oxygen, upon return to anaerobic conditions.

Here we interrogate this mechanistic proposal. This proposal requires that unactivated YfiD be able to bind cPFL, and we were able to demonstrate using ITC a discrete binding event between cPFL and YfiD with a K_D_ of 14 μM. Additionally, this proposal is based on the idea that the presence of cPFL enhances glycyl radical installation on YfiD through a rearrangement of the y-β1 strand away from the glycine to be activated. Consistent with this notion, we find that the presence of cPFL dramatically affected the installation of a glycyl radical on YfiD from no detectable activation in the absence of cPFL to wild-type PFL levels of activation in its presence (15–17 μM). By preventing robust activation of YfiD in the absence of oxygen-damaged, cleaved PFL, the cell is protected from a freely diffusing spare part protein with an exposed radical cofactor, and AdoMet and reducing equivalents are not wasted if this spare part protein is not needed, *i.e.*, if cPFL is not present in the cell.

Although the above mechanistic proposal provides an elegant explanation for how YfiD activation can be regulated by the presence or absence of cPFL, we are left trying to understand how binding of YfiD to cPFL may prompt movement of the y-β1 strand if y-β1’s binding site on cPFL is occupied by PFL’s β10 strand. The y-β1 strand of YfiD duplicates the β10 strand of PFL, which is not cleaved upon oxygen exposure (Fig. 3, *B* and *C*). We previously suggested that oxygen-induced cleavage of PFL could be followed by a proteolytic cleavage between positions 690 to 695, which would remove the β10 strand of PFL in addition to removing the remaining residues of the glycyl radical loop, allowing YfiD to bind (28). We previously reported that cPFL is subject to proteolysis between these residues 690 to 695 (28). Here we tested this hypothesis by generating a truncated PFL construct (tPFL) in which residues 696 to 759 are removed. We were expecting that YfiD would bind with higher affinity to tPFL than cPFL and that glycyl radical installation would be improved. However, we saw the exact opposite.

Our ITC data demonstrate that residues 696 to 733 of cPFL are critical for the binding of YfiD with no detectable binding observed for tPFL under the conditions used, *i.e.*, any enthalpic binding is much weaker than the heat of dilution and thus cannot be observed using ITC. Additionally, we found that the tPFL:YfiD complex could not be activated well (32-fold decrease in glycyl radical concentration from that of cPFL:YfiD). Once activated, tPFL complexes are just as active as wild-type, indicating that tPFL is properly folded and fully functional catalytically. The lower activation is consistent with the weaker binding of YfiD, and together they refute our previous hypothesis that residues 696 to 733 of cPFL must be truncated before YfiD can bind. Given that contacts made between YfiD’s y-β1 strand and the β9 strand of cPFL are likely critical for securing the glycyl radical loop in position in cPFL’s active site for catalysis (Fig. 3*C*) and that the glycyl radical loop of YfiD cannot bind to cPFL if half of the PFL glycyl radical loop is still in place, residues 696 to 733 of cPFL must move out of the active site. Thus, the new model for YfiD rescue involves *movement* of 696 to 733 of cPFL rather than *cleavage*.

That residues near the oxygen-cleavage site on cPFL may rearrange upon glycyl radical cleavage is not surprising. What is surprising is that facilitating the movement of those residues through truncation was detrimental instead of being beneficial or neutral. If movement of these residues occurs quickly and spontaneously following oxygen-induced cleavage, then we should have seen no effect as a result of truncation. However, we did see an effect, and it was a negative effect (weaker binding and lower activation). Such a negative effect suggests that residues 696 to 733 of cPFL constitute a recognition element for formation of the cPFL:YfiD complex.

The idea of a recognition element for YfiD is attractive and addresses one challenge of a spare-part-protein-rescue strategy, which is how to avoid putting a spare part on an enzyme that is too damaged for rescue. And, we suspect that cPFL may become “too damaged” quickly. In particular, we and others have observed that both PFL and cPFL are prone to proteolysis at multiple sites in addition to residue 695 (Fig. S5) (28, 35). Notably, residues 607 to 615 in PFL, which are readily truncated in the absence of YfiD repair, have been proposed to be important for CoA binding (36). Based on these findings with purified protein *in vitro*, we suspect that cPFL *in vivo* would be rapidly degraded in the cell if not quickly repaired by YfiD. There is a chemical logic to designing a fast route for degradation for an enzyme that is subject to damage under the conditions in which it is being expressed. Thus, YfiD rescue must be competing with cPFL degradation mechanisms. If cPFL is not repaired efficiently, it is cleared from the cell. Ideally, the system would be designed such that YfiD can recognize efficiently when PFL is cleaved and be ready for repair and also recognize when PFL is too extensively cleaved to be repaired.

A recognition element composed of residues 696 to 733, which are adjacent to the residue 734 cleavage site, is attractive as these residues are directly affected by the cleavage event. Also, these residues are the perfect signal that extensive cleavage has not occurred, as they would be the next set of residues to be truncated. Thus, if residues 696 to 733 are present, cPFL is sufficiently intact for successful repair by YfiD.

Our data also suggest that it might be the disordered N-terminal half of YfiD that is responsible for identifying cPFL’s 696 to 733 recognition element. First, our data indicate that residues 1 to 60 of YfiD are important for cPFL binding. In particular, the truncated YfiD variant, truncYfiD, that is missing residues 1 to 60, shows fourfold lower glycyl radical activation than full-length YfiD. Also, truncYfiD’s binding to cPFL is too weak to be measured by ITC. However, for the fraction of truncYfiD that does bind cPFL and can become activated, the enzyme activity level is similar to cPFL:YfiD and to PFL with only a slightly higher K_M_ (Table 1). Thus, the role of the disordered N-terminal half of YfiD appears to be in binding cPFL rather than for catalysis. We do not know where on cPFL the N-terminal half of YfiD binds, but the detrimental effect of removing residues 696 to 733 of PFL is lessened if residues 1 to 60 from YfiD are also missing (Fig. 5, **4** and **5**), indicating some degree of cross talk between these two regions. More work will need to be done to confirm or refute a direct binding event between 696 to 733 of cPFL and 1 to 60 of YfiD. At this point, however, we are confident that both regions are important for cPFL:YfiD complex formation.

**Figure 8:**
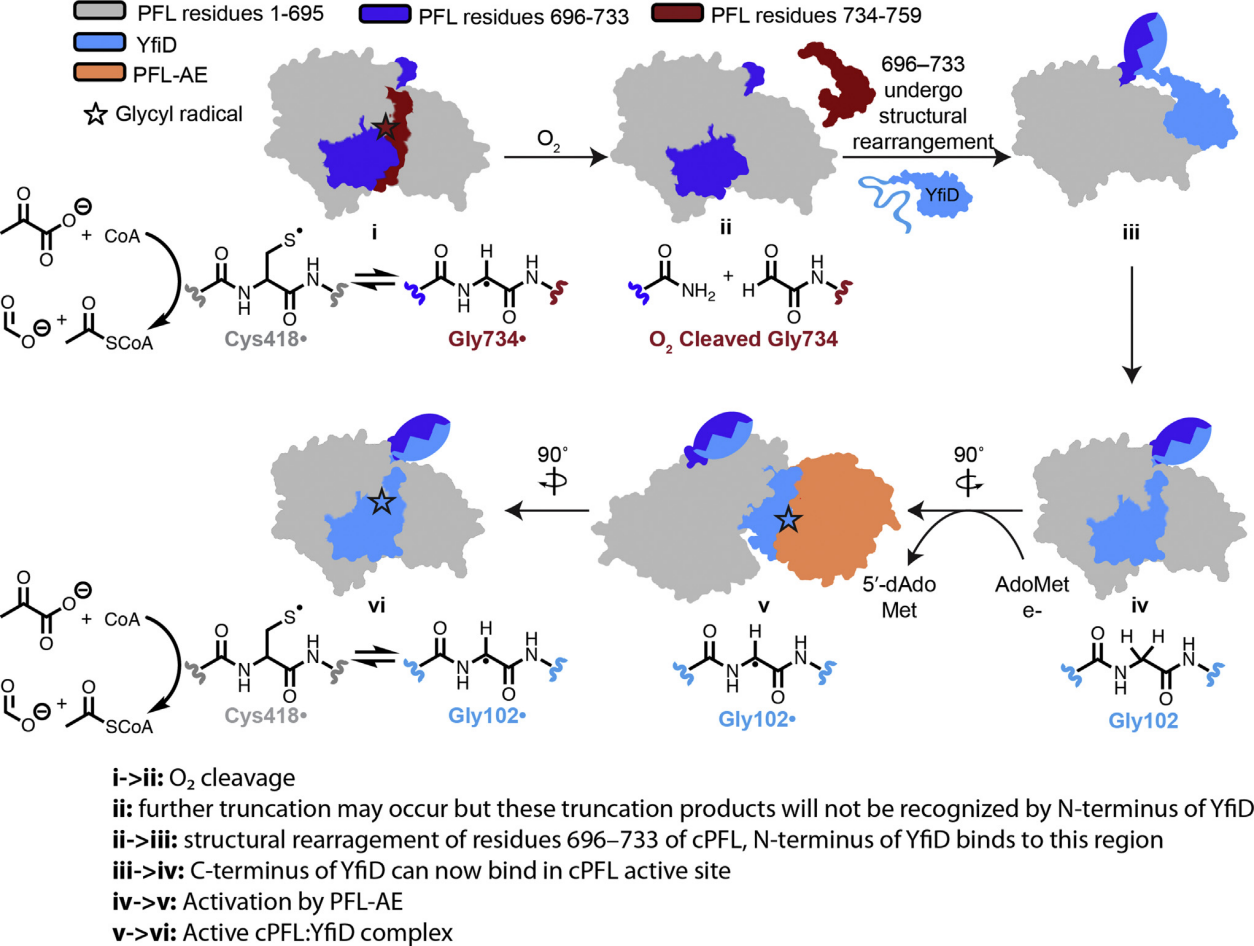
**Revised model for YfiD rescue of O**_**2**_**-damaged PFL.** Crystal structures of PFL and PFL-AE (PDB ID: 2PFL and 3CB8, respectively) and NMR structure of YfiD (PDB ID: 6OWR) were used to create cartoons. No structural data are available for any of the above protein complexes—cartoons of complexes were created by manually docking structures as previously described. Color coding is as follows: PFL residues 1 to 695 in *gray*, PFL residues 696 to 733 in *dark blue*, PFL residues 734 to 759 in *red*, PFL-AE in *orange*, YfiD in *light blue*.

Using these data, we have revisited and revised our previously proposed structure-based mechanism for YfiD repair of oxygen-damaged PFL (Fig. 8). In this revised model, oxygen-induced cleavage at position 734 (Fig. 8, i to ii) is no longer followed by proteolysis of residues 696 to 733 (Fig. 3*A*, ii to iii). Instead, we propose that residues 696 to 733 are a recognition element that promotes YfiD binding in an association event that is also facilitated by the N-terminal half of YfiD (Fig. 8, ii to iii). Such a recognition signal would serve to indicate that cPFL is sufficiently intact to justify use of the spare part protein YfiD in a repair process. Once the cPFL:YfiD complex is formed (Fig. 8, iv), the glycyl radical loop of YfiD can flip out of the active site, leaving the y-β1 strand anchored within cPFL, and the glycyl radical loop can bind to and be activated by PFL-AE without y-β1 strand interference (Fig. 8, v). Importantly, these latter steps of the mechanism are supported by spectroscopic, kinetic and ITC data. Lastly, once the glycyl radical has been installed, the glycyl radical loop can flip back into the active site of the complex and begin to catalyze formation of acetyl-CoA—a step in which the N-terminus of YfiD and residues 696 to 733 of cPFL are no longer needed (Fig. 8, vi). Incredibly, the activity of the repaired complex is as good as wild-type PFL if not ever so slightly better (Table 1).

Using multiple biophysical and biochemical techniques, we have been able to put forth a model for oxygen-damaged PFL rescue of activity by YfiD. Though spare part proteins have only been demonstrated biochemically for PFLs, it is possible that this rescue mechanism is common to the GRE family more broadly. Currently there are 2142 sequences in the InterPro family IPR011140, annotated as autonomous glycyl radical cofactors (GrcAs), a family of predicted YfiD-like proteins containing glycyl radical domains. Although the only characterized GrcAs thus far are YfiD/Y06I, and there is only experimental evidence for their repair of PFL, it is tempting to speculate that some of these putative spare part proteins could repair other GREs. All GREs use glycyl radical cofactors to initiate their chemistry and are all prone to the same type of oxidative damage. Fixing oxidatively damaged GREs with spare part proteins could be a broader mode of repair among anaerobes that rely on these critical metabolic enzymes.

## Experimental procedures

### Cloning of constructs

We received *yfid* in a pCAL-n-EK vector and *pfl-AE* in a pCAL-n-EK vector from the Broderick lab (18). An N-terminal His-tag and TEV cleavage site were added to the YfiD construct using the Q5 Site-Directed Mutagenesis Kit (New England Biolabs). The following primers were used for this insertion: forward primer, 5′-CCAACGACCGAGAATCTTTATTTTCAGGGATCCATGATTACAGGTATCCAG-3′, and reverse primer, 5′-ATCGTGATGGTGATGGTGATGGCTGCTAGCCATATGTATATCTCCTTCTTAAAGTTAAAC-3′. The His-tagged truncYfiD construct was previously reported (28).

The PFL and cPFL constructs were synthesized and cloned into pET21d at NcoI and XhoI restriction sites by GenScript. An N-terminal His-tag and TEV cleavage site were added to each of the constructs. The tPFL construct was made by introducing a stop codon at residue E696 in the cPFL plasmid. The stop codon was added using the NEB Mutagenesis Kit and the following primers: forward primer, 5′-CTTCCACCACTAAGCATCCATC-3′, and reverse primer, 5′- TAACCATCCATCAGACCAG-3′. All primers were designed using NEBaseChanger. All mutagenesis experiments were confirmed through Sanger sequencing by GENEWIZ, Inc.

### Expressions and purifications

All *pfl* and *yfid* mutants were transformed into T7 Express cells (New England BioLabs) and were expressed as follows. Starter cultures were inoculated from glycerol stocks and grown overnight in LB containing 100 μg/ml ampicillin (GoldBio) at 37 °C at 220 rpm. Expression cultures were inoculated with 5 ml of starter culture per 1 l of LB containing 100 μg/ml ampicillin. Expression cultures were grown at 37 °C at 220 rpm to an OD_600_ = 0.6 to 0.8, at which point they were induced with 1 mM IPTG (GoldBio). Induced cultures were expressed for 3 h at 37 °C at 220 rpm. Cells were pelleted by centrifugation and stored at −80 °C until lysis.

For lysis of cells containing YfiD variants, cell paste from 1 l of culture was resuspended in 10 ml lysis buffer (lysis buffer: 20 mM HEPES pH 7.2, 10 mM MgCl_2_, 300 mM NaCl, 5% glycerol, 1% Triton X-100), with an EDTA-free protease inhibitor pellet (cOmplete, Roche Diagnostics), lysozyme (1 mg lysozyme/ml buffer, Sigma Aldrich), and 1 μl benzonase (EMD Millipore). Cells were agitated on a rotary mixer at 4 °C for 60 min, after which cells were sonicated for 4 × 2 min cycles of 2 s on and 2 s off at 60% power (Branson Digital Sonifier). Lysate was clarified by centrifugation for 45 min at 28,000*g* and subsequently filtered (0.22 μm) before purification. For lysis of cells containing PFL variants, a gentler lysis protocol was developed to minimize further truncation. Cell paste from 1 l of culture was resuspended in 10 ml lysis buffer (lysis buffer: 20 mM HEPES pH 7.2, 10 mM MgCl_2_, 5% glycerol, 1% Triton X-100), with an EDTA-free protease inhibitor pellet (cOmplete, Roche Diagnostics), lysozyme (1 mg lysozyme/ml buffer, Sigma Aldrich), and 1 μl benzonase (EMD Millipore). After resuspension, cells were sonicated for 1 × 2 min cycles of 2 s on and 2 s off at 60% power (Branson 450 Digital Sonifier). Lysate was clarified by centrifugation for 45 min at 28,000*g* and subsequently filtered (0.22 μm) before purification. PFL variants were always handled at 4 °C unless otherwise noted to reduce further truncation.

His-tagged YfiD variants were purified on gravity-packed Ni-NTA resin using buffers containing 20 mM HEPES pH 7.2 and imidazole increasing from 10 to 250 mM. Pure YfiD fractions were pooled and desalted using a HiPrep 26/10 Desalting column (GE) into 20 mM HEPES pH 7.2 buffer. His-tagged TEV protease was added to purified YfiD variants at a ratio of 10:1 (YfiD:TEV protease, w/w) to remove the N-terminal Histag. The reaction was gently mixed and left at 4 °C for ∼24 h (or until >80% completion as determined by SDS-PAGE) without agitation. The reaction mixture was purified on Ni-NTA resin as detailed above. Fractions containing pure YfiD with the Histag removed were pooled and desalted using a HiPrep 26/10 Desalting column (GE) into 20 mM HEPES pH 7.2 buffer. YfiD variants were concentrated using an Amicon spin cell concentrator with a 3 kDa membrane MWCO, aliquoted, and flash frozen.

PFL variants were purified on gravity-packed TALON resin using buffers containing 50 mM HEPES pH 7.2, 300 mM NaCl, and imidazole increasing from 0 to 100 mM. Pure fractions were pooled, desalted using a HiPrep 26/10 Desalting column (GE) into 20 mM HEPES pH 7.2 buffer, and concentrated using a Millipore 50 kDa centrifugal filter to ∼30 mg/ml, aliquoted, and flash frozen.

PFL-AE was expressed and purified similarly to previously published protocols (18). The PFL-AE construct was transformed into BL21pLysS cells for expression. Starter cultures were inoculated from a glycerol stock and grown overnight in LB containing 50 μg/ml ampicillin at 37 °C at 220 rpm. Expression cultures were inoculated with 15 ml of starter culture per 1.5 l of LB containing 50 μg/ml ampicillin. Expression cultures were grown at 37 °C at 220 rpm to an OD_600_ = 0.3, at which point D-glucose was added (0.5% w/v). Cultures continued to grow at 37 °C at 220 rpm to an OD_600_ = 0.8, at which point 300 mM L-cysteine and 300 mM (NH_4_)_2_Fe(SO_4_)_2_ were added, and cultures were induced with 0.25 mM IPTG. Induced cultures were expressed for a total of 5 h at 30 °C at 220 rpm. Two hours after inducing overexpression, another 300 mM of L-cysteine and (NH_4_)_2_Fe(SO_4_)_2_ were added to the cultures. After expression, cultures were sparged with argon overnight at 4 °C to remove oxygen. Cultures were added to sealable centrifugation buckets in a Coy anaerobic chamber, and cells were pelleted by centrifugation and stored at −80 °C until lysis.

Cell lysis and protein purification of PFL-AE were performed anaerobically in an MBraun chamber. All buffers were sparged with argon before use. For lysis of PFL-AE cells, frozen cell paste from 1.5 l of culture was cycled into an anaerobic MBraun chamber and resuspended in 5 ml lysis buffer (lysis buffer: 50 mM Tris pH 7.5, 10 mM MgCl_2_, 100 mM NaCl, 5% glycerol, 1% Triton X-100, 7 mM DTT) with an EDTA-free protease inhibitor pellet (cOmplete, Roche Diagnostics), lysozyme (0.5 mg lysozyme/ml buffer, Sigma Aldrich), and 1 μl benzonase (EMD Millipore). Cells were resuspended by mashing cell paste with a spatula. Resuspended cells were incubated for 60 min at 4 °C, after which cells were sonicated for a 1 × 2 min cycle of 2 s on and 15 s off at an amplitude of 10 (Qsonica). Lysate was clarified by centrifugation for 45 min at 28,000*g* and subsequently filtered (0.22 μm) before purification.

PFL-AE was purified by loading 5 ml of clarified lysate onto a HiLoad 16/60 Superdex 75 prep grade (GE) column and using an isocratic method with buffer composed of 50 mM Tris pH 7.5, 100 mM NaCl, and 1 mM DTT. PFL-AE eluted as a monomer, and the Fe content of purified PFL-AE was determined to be 2.9 Fe per monomer (37, 38) which is consistent with previous reports (18).

Protein concentrations were determined using absorbance at 280 nm on a Nanodrop 2000c spectrophotometer (Thermo Scientific) using the molar extinction coefficients 85,910 M^−1^ cm^−1^ for PFL; 84,630 M^−1^ cm^−1^ for cPFL; 83,350 M^−1^ cm^−1^ for tPFL; 2560 M^−1^ cm^−1^ for truncYfiD; 10,810 M^−1^ cm^−1^ for YfiD; and 39,420 M^−1^ cm^−1^ for PFL-AE.

### Photoreduction/activation reactions for glycyl radical quantitation and kinetic analysis

In an MBraun anaerobic chamber, wtPFL, cPFL, tPFL, YfiD, and truncYfiD were diluted to 200 μM for each component (note that PFL is a dimer in solution; however, calculations were done such that PFL concentration is given in number of available binding sites for YfiD, which is two per dimer) with 20 mM HEPES pH 7.2 to a final volume of 150 μl. Pyruvate (final conc. 10 mM), PFL-AE (final conc. 5 μM), AdoMet (final conc. 0.2 mM, gift from Vahe Bandarian, synthesized as described in Young and Bandarian (39)) and 5-deazariboflavin (final conc. 50 μM, Santa Cruz Biotechnology) were added to each reaction. Activation buffer (50 mM Tris pH 7.4, 100 mM NaCl, 10 mM DTT) was added to each reaction for a final volume of 300 μl. The activations were mixed by pipetting and placed in a cooled water bath that is kept below 30 °C. The activations were illuminated using a 500 W halogen lamp for 15 to 30 min. A small aliquot of each reaction (20–40 μl) is kept in the dark and anaerobic at 4 °C for kinetic analysis, and the remaining samples are anaerobically frozen in liquid nitrogen for EPR spectroscopy.

### Quantification of glycyl radical using EPR spectroscopy

EPR spectra were collected in a Bruker EMX-Plus spectrometer at 80 K with a Bruker/ColdEdge 4 K waveguide cryogen-free cryostat. Xenon 1.1b.155 software was used to collect and process spectra. Spectra were recorded at 9.37 GHz with a modulation amplitude of 3 G, microwave power of 1.26 μW, and a 100 kHz modulation frequency. A center field of 3350 G, a sweep time of 21 s, and a sweep width of 200 G were used. Each spectrum shown is an average of 10 scans. All spectra used in Figure 5 were collected the same day for activation reactions conducted in triplicate. Potassium nitrosodisulfonate (Fremy’s salt, Sigma Aldrich) was used as a standard. The double integrals of each spectrum were calculated using Xenon software and compared with the double integrals obtained from Fremy’s standard to obtain concentrations of glycyl radical. The concentration of glycyl radical was used to calculate k_cat_ in kinetic analysis.

### Coupled assays for kinetic analysis

A previously reported coupled assay was used to determine kinetic parameters for PFL and PFL:YfiD complexes (11). Briefly, an equilibrium of NAD reduction to NADH is established for citrate synthase (acetyl-CoA and oxaloacetate to citrate and CoA) and malic acid dehydrogenase (malate and NAD to oxaloacetate and NADH). A known concentration of activated PFL or PFL:YfiD is added to the reaction and the equilibrium shifts to form more NADH as acetyl-CoA is produced by PFL or PFL:YfiD. This formation of NADH is measured by UV-vis spectroscopy and used to calculate initial velocity curves (11). Activation of PFL:YfiD complexes, quantification of glycyl radical using EPR spectroscopy, and assays for kinetic analysis were always performed on the same day so as to minimize glycyl radical degradation over time. Assay buffer (150 mM Tris pH 8.5, 10 mM L-malate, 10 mM pyruvate, 3 mM NAD) was always made fresh the day of experiments. Assay buffer was sparged with argon and brought into an anaerobic chamber. Inside the chamber, citrate synthase (6 U per reaction, Sigma Aldrich) and malic acid dehydrogenase (14 U per reaction, Sigma Aldrich) were added to the assay buffer. Coenzyme A (Sigma Aldrich) was added as a solution in water to final concentrations ranging from 2.5 to 400 μM per reaction. The activated PFL or PFL:YfiD mixture was added to initiate the reaction and immediately pipetted to mix. Data were collected on an Ocean Optics Spectrometer at 366 nm to measure absorbance of NADH. Initial velocity curves were conducted in triplicate for each CoA concentration at 21 °C and plotted using Prism nonlinear regression software (Fig. 6) to calculate K_M_ and V_max_ for each complex (Table 1). EPR spectroscopy was used to measure glycyl radical content for PFL and PFL:YfiD complexes (see above), and the final concentrations of radical in reactions were used as E_tot_. V_max_ and E_tot_ were used to calculate k_cat_ (Table 1).

## Isothermal titration calorimetry

All data were collected on a MicroCal iTC200. For the cPFL:YfiD complex, cPFL (205.1 μl of 224 μM cPFL) was loaded into the sample chamber and YfiD (100 μl of 2.129 mM) was loaded into the syringe. Final concentrations after 20 injections were 186 and 361 μM for cPFL and YfiD, respectively. For the cPFL:truncYfiD complex, cPFL (205.1 μl of 173 μM cPFL) was loaded into the sample chamber and truncYfiD (100 μl of 1.73 mM) was loaded into the syringe. Final concentrations after 20 injections were 143 and 294 μM for cPFL and truncYfiD, respectively. For the tPFL:YfiD complex, tPFL (205.1 μl of 224 μM tPFL) was loaded into the sample chamber and YfiD (100 μl of 2.1 mM) was loaded into the syringe. Final concentrations after 20 injections were 186 and 356 μM for tPFL and YfiD, respectively. ITC experiments are commonly conducted at higher than physiological protein concentrations, which for PFL have been estimated to be 20 μM (40), in order to be able to measure the heat change. The parameters for all isotherms were set as follows: number of injections = 20, cell temperature = 25 °C, reference power = 10 μcal/s, initial delay = 60 s, stirring speed 300 rpm, injection volume = 2.0 μl, duration = 4 s, spacing = 180 s, filter period = 5 s. The first injection volume was set to 0.4 μl, and this data point was removed from all isotherms per manufacturer’s recommendations. Resulting data were analyzed and fit using MicroCal Analysis software.

## Data availability

All data are contained within the manuscript.

## Supporting information

This article contains supporting information.

## Conflicts of interest

The authors declare that they have no conflicts of interest with the contents of this article.

## References

[1] Backman L.R.F., Funk M.A., Dawson C.D., Drennan C.L. (2017). New tricks for the glycyl radical enzyme family. Crit. Rev. Biochem. Mol. Biol..

[2] Sun X., Ollagnier S., Schmidt P.P., Atta M., Mulliez E., Lepape L., Eliasson R., Graslund A., Fontecave M., Reichard P., Sjoberg B.M. (1996). The free radical of the anaerobic ribonucleotide reductase from *Escherichia coli* is at glycine 681. J. Biol. Chem..

[3] Leuthner B., Leutwein C., Schulz H., Horth P., Haehnel W., Schiltz E., Schagger H., Heider J. (1998). Biochemical and genetic characterization of benzylsuccinate synthase from Thauera aromatica: A new glycyl radical enzyme catalysing the first step in anaerobic toluene metabolism. Mol. Microbiol..

[4] Levin B.J., Balskus E.P. (2018). Discovering radical-dependent enzymes in the human gut microbiota. Curr. Opin. Chem. Biol..

[5] Levin B.J., Huang Y.Y., Peck S.C., Wei Y., Martinez-Del Campo A., Marks J.A., Franzosa E.A., Huttenhower C., Balskus E.P. (2017). A prominent glycyl radical enzyme in human gut microbiomes metabolizes trans-4-hydroxy-l-proline. Science.

[6] Backman L.R., Huang Y.Y., Andorfer M.C., Gold B., Raines R.T., Balskus E.P., Drennan C.L. (2020). Molecular basis for catabolism of the abundant metabolite trans-4-hydroxy-L-proline by a microbial glycyl radical enzyme. Elife.

[7] Peck S.C., Denger K., Burrichter A., Irwin S.M., Balskus E.P., Schleheck D. (2019). A glycyl radical enzyme enables hydrogen sulfide production by the human intestinal bacterium Bilophila wadsworthia. Proc. Natl. Acad. Sci. U. S. A..

[8] Xing M., Wei Y., Zhou Y., Zhang J., Lin L., Hu Y., Hua G., Nanjaraj Urs A.N., Liu D., Wang F., Guo C., Tong Y., Li M., Liu Y., Ang E.L. (2019). Radical-mediated C-S bond cleavage in C2 sulfonate degradation by anaerobic bacteria. Nat. Commun..

[9] Dawson C.D., Irwin S.M., Backman L.R.F., Le C., Wang J.X., Vennelakanti V., Yang Z., Kulik H.J., Drennan C.L., Balskus E.P. (2021). Molecular basis of C-S bond cleavage in the glycyl radical enzyme isethionate sulfite-lyase. Cell Chem. Biol..

[10] Beller H.R., Rodrigues A.V., Zargar K., Wu Y.W., Saini A.K., Saville R.M., Pereira J.H., Adams P.D., Tringe S.G., Petzold C.J., Keasling J.D. (2018). Discovery of enzymes for toluene synthesis from anoxic microbial communities. Nat. Chem. Biol..

[11] Knappe J., Blaschkowski H.P., Grobner P., Schmitt T. (1974). Pyruvate formate-lyase of *Escherichia coli*: The acetyl-enzyme intermediate. Eur. J. Biochem..

[12] Knappe J., Wagner A.F. (1995). Glycyl free radical in pyruvate formate-lyase: Synthesis, structure characteristics, and involvement in catalysis. Methods Enzymol..

[13] Liu J.Z., Xu W., Chistoserdov A., Bajpai R.K. (2016). Glycerol dehydratases: Biochemical structures, catalytic mechanisms, and industrial applications in 1,3-propanediol production by naturally occurring and genetically engineered bacterial strains. Appl. Biochem. Biotechnol..

[14] Jager C.M., Croft A.K. (2018). Anaerobic radical enzymes for biotechnology. Chembioeng Rev..

[15] Zelcbuch L., Lindner S.N., Zegman Y., Vainberg Slutskin I., Antonovsky N., Gleizer S., Milo R., Bar-Even A. (2016). Pyruvate formate-lyase enables efficient growth of *Escherichia coli* on acetate and formate. Biochemistry.

[16] Rabus R., Boll M., Heider J., Meckenstock R.U., Buckel W., Einsle O., Ermler U., Golding B.T., Gunsalus R.P., Kroneck P.M., Kruger M., Lueders T., Martins B.M., Musat F., Richnow H.H. (2016). Anaerobic microbial degradation of hydrocarbons: From enzymatic reactions to the environment. J. Mol. Microbiol. Biotechnol..

[17] Conradt H., Hohmann-Berger M., Hohmann H.P., Blaschkowski H.P., Knappe J. (1984). Pyruvate formate-lyase (inactive form) and pyruvate formate-lyase activating enzyme of *Escherichia coli*: Isolation and structural properties. Arch. Biochem. Biophys..

[18] Henshaw T.F., Cheek J., Broderick J.B. (2000). The [4Fe-4S]^1+^ cluster of pyruvate formate-lyase activating enzyme generates the glycyl radical on pyruvate formate-lyase: EPR-detected single turnover. J. Am. Chem. Soc..

[19] Vey J.L., Yang J., Li M., Broderick W.E., Broderick J.B., Drennan C.L. (2008). Structural basis for glycyl radical formation by pyruvate formate-lyase activating enzyme. Proc. Natl. Acad. Sci. U. S. A..

[20] Peng Y., Veneziano S.E., Gillispie G.D., Broderick J.B. (2010). Pyruvate formate-lyase, evidence for an open conformation favored in the presence of its activating enzyme. J. Biol. Chem..

[21] Wagner A.F., Frey M., Neugebauer F.A., Schafer W., Knappe J. (1992). The free radical in pyruvate formate-lyase is located on glycine-734. Proc. Natl. Acad. Sci. U. S. A..

[22] Licht S., Gerfen G.J., Stubbe J. (1996). Thiyl radicals in ribonucleotide reductases. Science.

[23] Shisler K.A., Broderick J.B. (2014). Glycyl radical activating enzymes: Structure, mechanism, and substrate interactions. Arch. Biochem. Biophys..

[24] Hanson A.D., McCarty D.R., Henry C.S., Xian X., Joshi J., Patterson J.A., Garcia-Garcia J.D., Fleischmann S.D., Tivendale N.D., Millar A.H. (2021). The number of catalytic cycles in an enzyme's lifetime and why it matters to metabolic engineering. Proc. Natl. Acad. Sci. U. S. A..

[25] Reddy S.G., Wong K.K., Parast C.V., Peisach J., Magliozzo R.S., Kozarich J.W. (1998). Dioxygen inactivation of pyruvate formate-lyase: EPR evidence for the formation of protein-based sulfinyl and peroxyl radicals. Biochemistry.

[26] Zhang W., Wong K.K., Magliozzo R.S., Kozarich J.W. (2001). Inactivation of pyruvate formate-lyase by dioxygen: Defining the mechanistic interplay of glycine 734 and cysteine 419 by rapid freeze-quench EPR. Biochemistry.

[27] Wagner A.F., Schultz S., Bomke J., Pils T., Lehmann W.D., Knappe J. (2001). YfiD of *Escherichia coli* and Y06I of bacteriophage T4 as autonomous glycyl radical cofactors reconstituting the catalytic center of oxygen-fragmented pyruvate formate-lyase. Biochem. Biophys. Res. Commun..

[28] Bowman S.E.J., Backman L.R.F., Bjork R.E., Andorfer M.C., Yori S., Caruso A., Stultz C.M., Drennan C.L. (2019). Solution structure and biochemical characterization of a spare part protein that restores activity to an oxygen-damaged glycyl radical enzyme. J. Biol. Inorg. Chem..

[29] Ben-Zvi O., Grinberg I., Orr A.A., Noy D., Tamamis P., Yacoby I., Adler-Abramovich L. (2021). Protection of oxygen-sensitive enzymes by peptide hydrogel. ACS Nano.

[30] Mahidhara G., Burrow H., Sasikala C., Ramana C.V. (2019). Biological hydrogen production: Molecular and electrolytic perspectives. World J. Microbiol. Biotechnol..

[31] Lu Y., Koo J. (2019). O2 sensitivity and H2 production activity of hydrogenases-A review. Biotechnol. Bioeng..

[32] Wyborn N.R., Messenger S.L., Henderson R.A., Sawers G., Roberts R.E., Attwood M.M., Green J. (2002). Expression of the *Escherichia coli* yfiD gene responds to intracellular pH and reduces the accumulation of acidic metabolic end products. Microbiology.

[33] Sauter M., Sawers R.G. (1990). Transcriptional analysis of the gene encoding pyruvate formate-lyase-activating enzyme of *Escherichia coli*. Mol. Microbiol..

[34] Frey M., Rothe M., Wagner A.F., Knappe J. (1994). Adenosylmethionine-dependent synthesis of the glycyl radical in pyruvate formate-lyase by abstraction of the glycine C-2 pro-S hydrogen atom. Studies of [2H]glycine-substituted enzyme and peptides homologous to the glycine 734 site. J. Biol. Chem..

[35] Rodel W., Plaga W., Frank R., Knappe J. (1988). Primary structures of *Escherichia coli* pyruvate formate-lyase and pyruvate-formate-lyase-activating enzyme deduced from the DNA nucleotide sequences. Eur. J. Biochem..

[36] Hanzevacki M., Banhatti R.D., Condic-Jurkic K., Smith A.S., Smith D.M. (2019). Exploring reactive conformations of coenzyme A during binding and unbinding to pyruvate formate-lyase. J. Phys. Chem. A..

[37] Fish W.W. (1988). Rapid colorimetric micromethod for the quantitation of complexed iron in biological samples. Methods Enzymol..

[38] Stookey L.L. (1970). Ferrozine - a new spectrophotometric reagent for iron. Anal. Chem..

[39] Young A.P., Bandarian V. (2011). Pyruvate is the source of the two carbons that are required for formation of the imidazoline ring of 4-demethylwyosine. Biochemistry.

[40] Crain A.V., Broderick J.B. (2014). Pyruvate formate-lyase and its activation by pyruvate formate-lyase activating enzyme. J. Biol. Chem..

