# Supplementary material for: Rescuing activity of oxygen-damaged pyruvate formate-lyase by a spare part protein
Source: J Biol Chem. 2021 Nov 18;297(6):101423. doi: 10.1016/j.jbc.2021.101423 (PMC8683613; doi:10.1016/j.jbc.2021.101423)
Supplement: Figures S1–S5 and Table S1 [file mmc1.docx]

**Supporting Information**

**Figure S1: Standard curve for quantification of glycyl radical by EPR.** Fremy’s standards were used to quantify the amount of glycyl radical on PFL and PFL:YfiD complexes. New standard curves were obtained the same day as PFL and PFL:YfiD experiments were conducted to account for day to day variation. The standard curve used to calculate glycyl radical concentrations in Figure 3 and Table S1 is shown above.

**Table S1: Triplicate data used in Figure 3.** To obtain the data shown in Figure 3, three activation reactions for each PFL and each complex were conducted in parallel. EPR spectra were collected for each reaction, and a standard curve of Fremy’s salt (Figure S2) was used to convert the double integral of each glycyl radical signal to concentration of glycyl radical. The calculated concentrations of glycyl radical for these 15 activation reactions are shown here in μM.

**
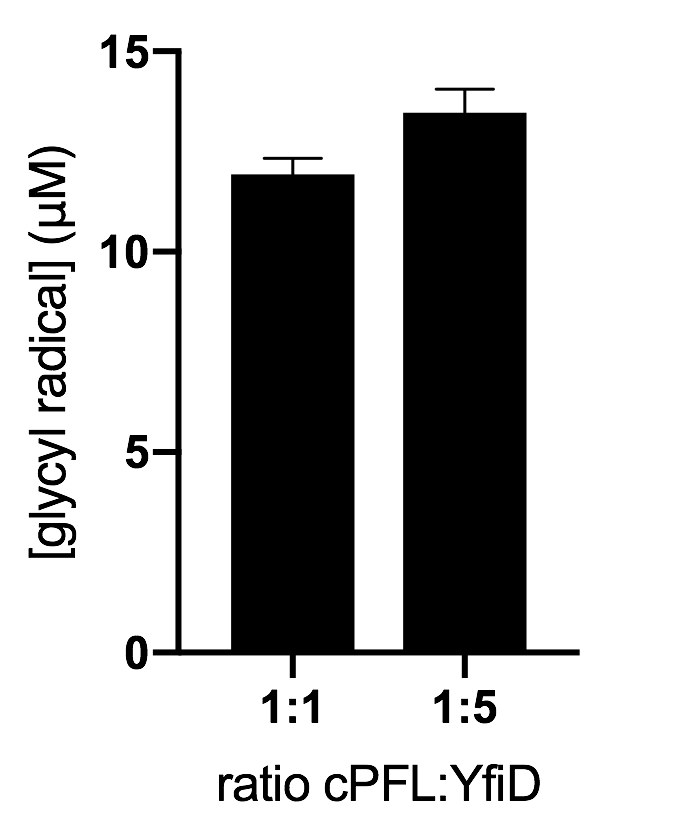
**

**Figure S2: Effect of increasing ratio of YfiD to PFL variants in activation reactions.** In an MBraun anaerobic chamber, cPFL or tPFL (100 μM final conc.) and YfiD (100 μM final conc. for 1:1 ratio and 500 μM final conc. for 1:5 ratio) were diluted with 20 mM HEPES pH 7.2 to a final volume of 150 μL. Pyruvate (final conc. 10 mM), PFL-AE (final conc. 5 μM), AdoMet (final conc. 0.2 mM), and 5-deazariboflavin (final conc. 50 μM) were added to each reaction. Activation buffer (50 mM Tris pH 7.4, 100 mM NaCl, 10 mM DTT) was added to each reaction for a final volume of 300 μL. Each reaction was performed in triplicate. The activations were mixed by pipetting and placed in a cooled water bath that is kept below 30 ˚C. The activations were illuminated using a 500 W halogen lamp for 15–30 minutes. EPR spectroscopy was used to quantify glycyl radical content.

**Figure S3: ITC controls.** To assess YfiD heat of dilution and ensure no large effects from potential oligomerization state changes were observed, ITC controls were performed. Isotherms were collected by titrating YfiD (left, 2.129 mM, 361 μM final conc.) or truncYfiD (right, 1.878 mM, 319 μM final conc.) into a cell of matched buffer. The cell contained only buffer; however, for processing, cell concentrations were set to 0.224 mM and 0.188 mM, respectively, for comparison to isotherms shown in Figure 4 (i.e. cell concentrations set so that the x-axes are comparable). Two data points from the YfiD isotherm on the left were removed due to problems with those particular injections (most likely trapped air bubbles). The parameters for all isotherms were set as follows: number of injections = 20, cell temperature = 25 ˚C, reference power = 10 μcal/s, initial delay = 60 s, stirring speed 300 rpm, injection volume = 2 μL, duration = 4 s, spacing = 180 s, filter period = 5 s. The first injection volume was set to 0.4 μL, and this data point was removed from all isotherms per manufacturer’s recommendations. Resulting data were analyzed and fit using MicroCal Analysis software.


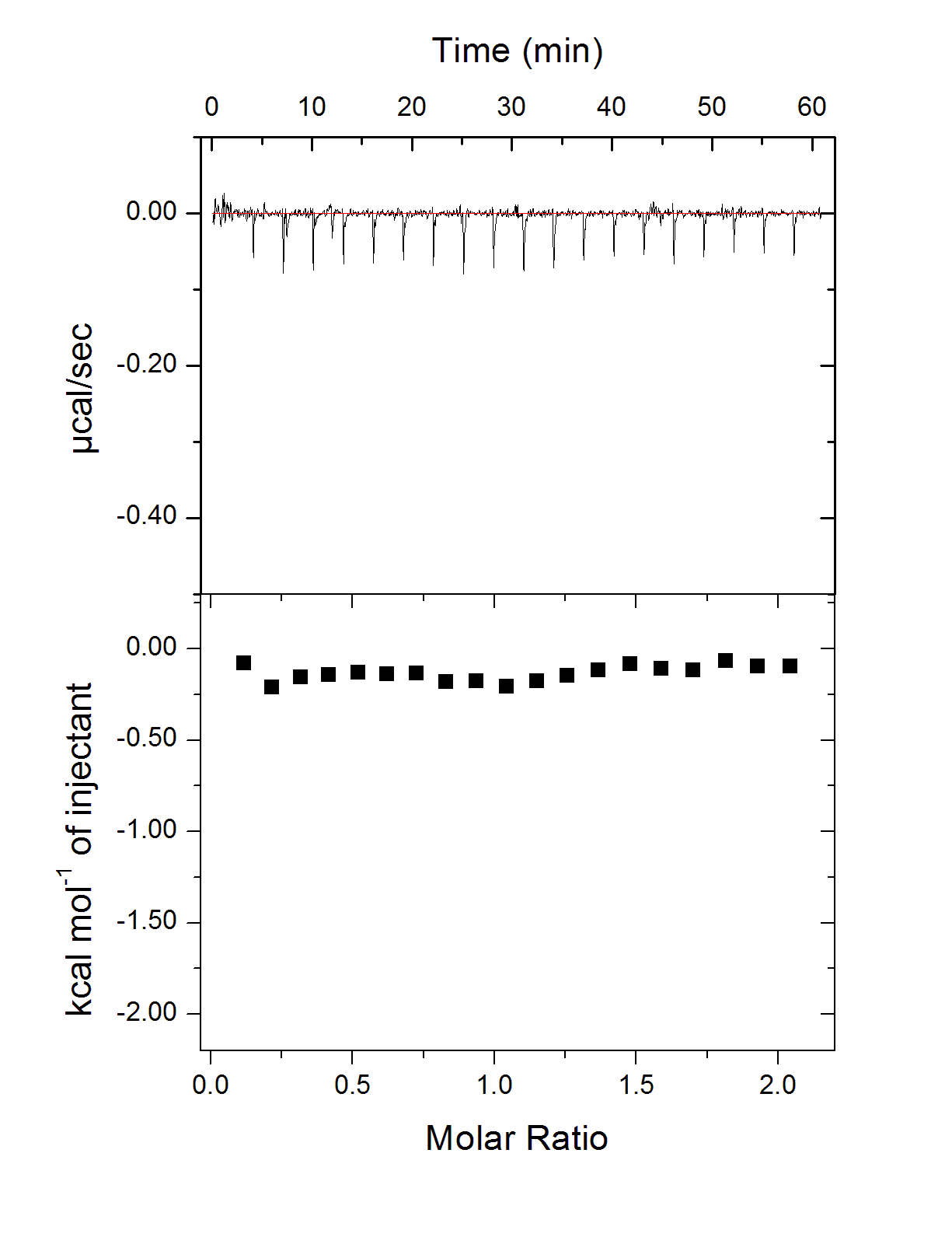

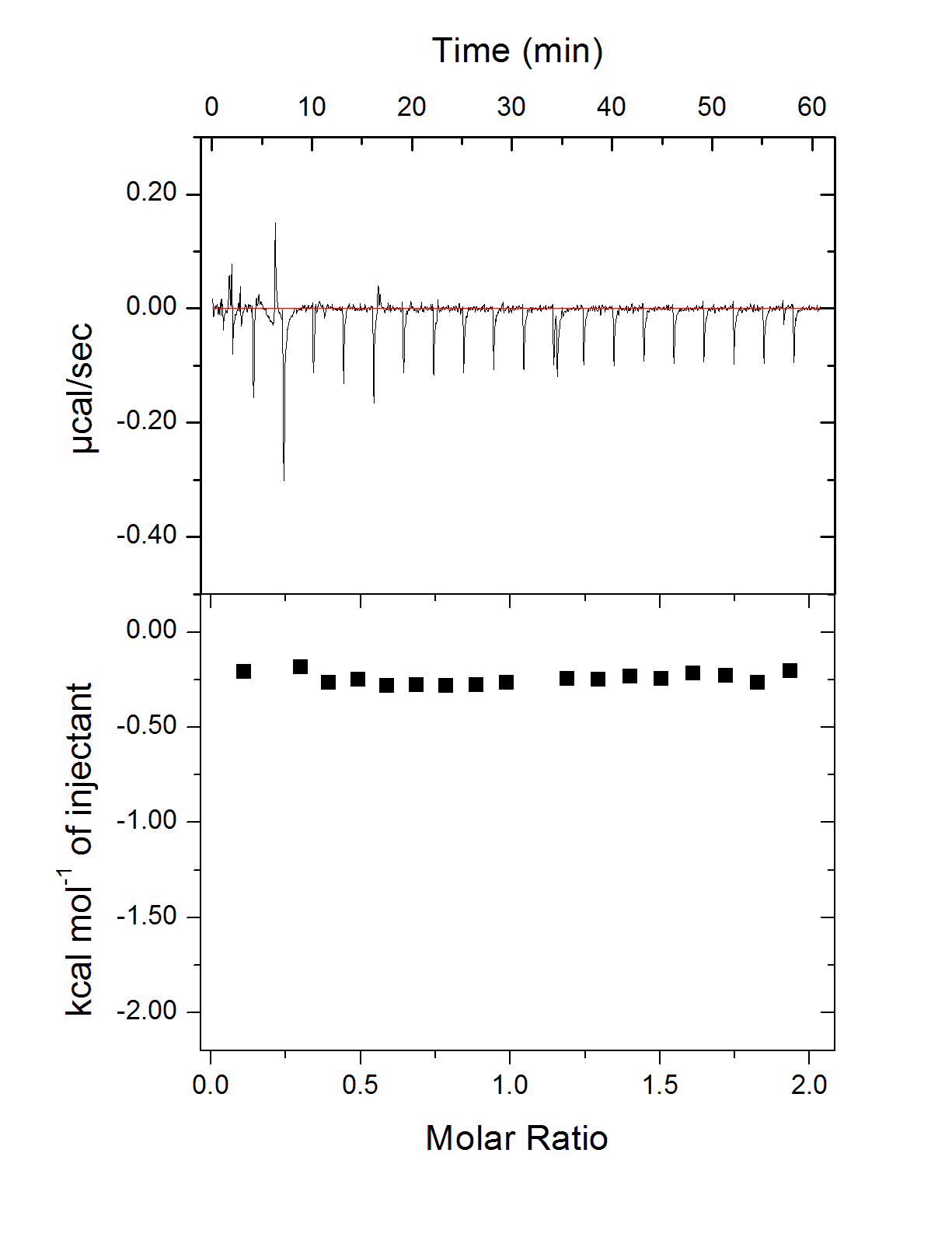


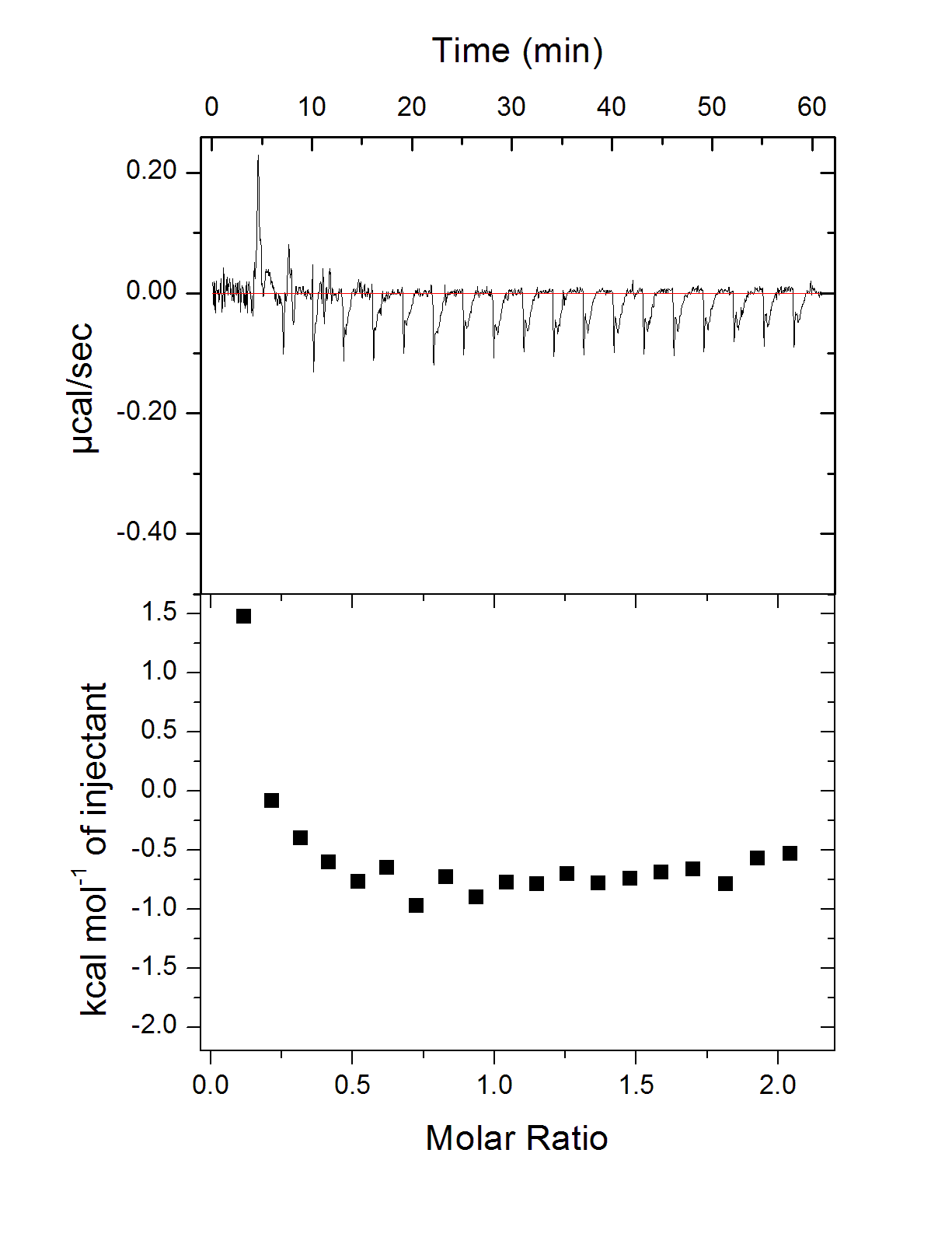


**Figure S4: ITC of truncYfiD titrated into tPFL.** truncYfiD (1.878 mM, 319 μM final conc.) was titrated into a cell containing tPFL (188 μM, 156 μM final conc.). The parameters for all isotherms were set as follows: number of injections = 20, cell temperature = 25 ˚C, reference power = 10 μcal/s, initial delay = 60 s, stirring speed 300 rpm, injection volume = 2 μL, duration = 4 s, spacing = 180 s, filter period = 5 s. The first injection volume was set to 0.4 μL, and this data point was removed from all isotherms per manufacturer’s recommendations. Resulting data were analyzed and fit using MicroCal Analysis software.


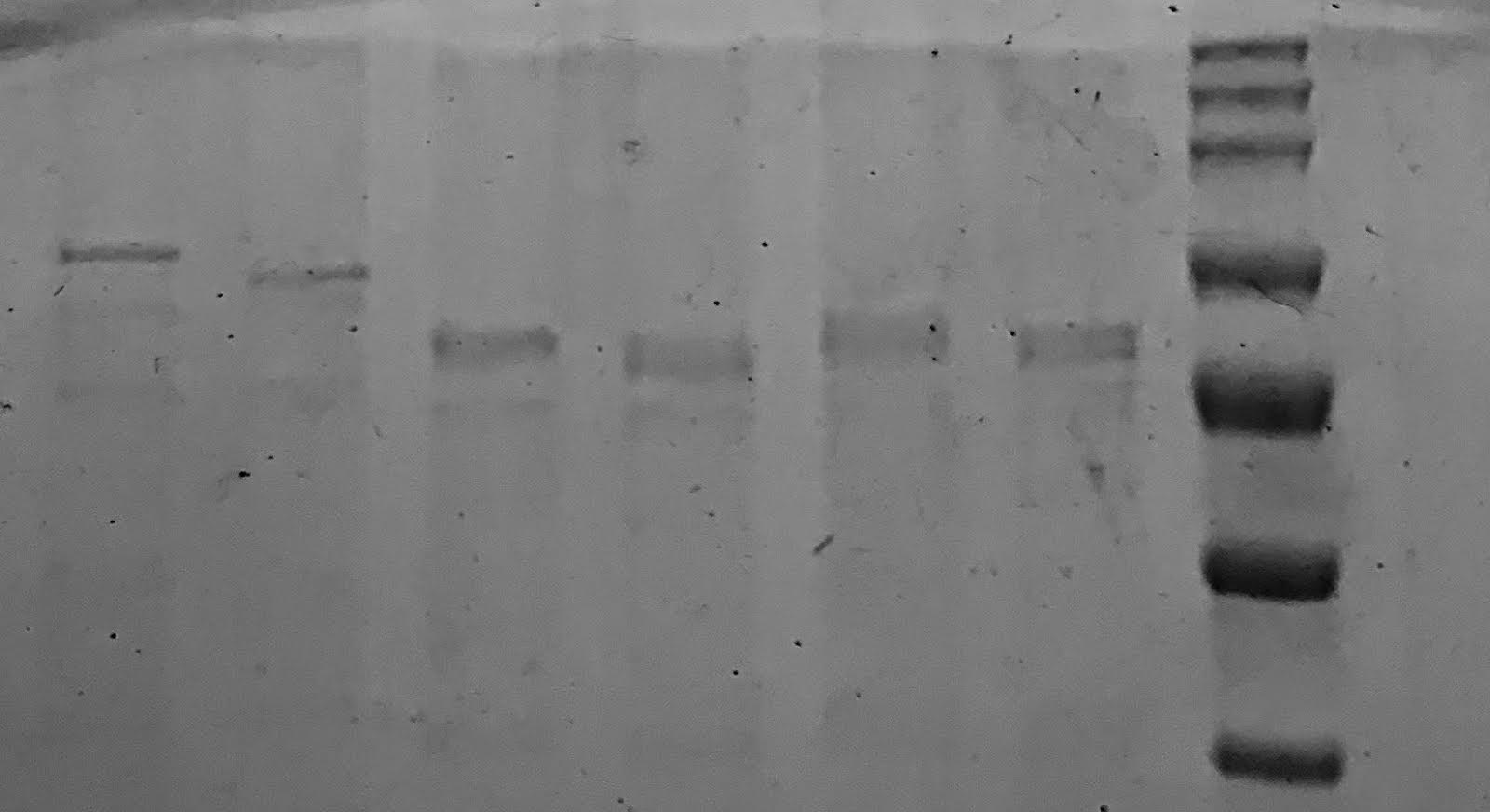


**wtPFL**

**cPFL**

**tPFL**

Uncharacterized truncation products

beyond tPFL

}

**1**

**2**

**3**

**4**

–245

–190

–135

KDa

–100

–80

–58

–46

**Figure S5: SDS-PAGE showing truncation of wtPFL and cPFL after one week.** Lanes 1 and 2 are full length standards of wtPFL and cPFL, respectively. tPFL is also indicated on the gel by an arrow. An aliquot of purified wtPFL (lane 3) and cPFL (lane 4) was left in 20 mM HEPES buffer at room temperature for one week. After one week, wtPFL and cPFL have both truncated to fragments lower than tPFL.
